# OA02.02. Effect of MBSR and psychological state on inflammatory markers in HIV positive adults

**DOI:** 10.1186/1472-6882-12-S1-O6

**Published:** 2012-06-12

**Authors:** E Weston, P Moran, M Acree, J Moskowitz, M Kemeny, E Elissa, P Bacchetti, K Barrows, S Deeks, F Hecht

**Affiliations:** 1 University of California, San Francisco (UCSF), Osher Center for Integrative Med, San Francisco, USA; 2 Department of Psychiatry, UCSF, San Francisco, USA; 3 Department of Epidemiology and Biostatistics, UCSF, San Francisco, USA; 4 Department of Medicine, UCSF, San Francisco, USA

## Purpose

HIV induces a pro-inflammatory response that is linked to increased morbidity and mortality. Stress and depression have been associated with elevated inflammation. We sought to test whether Mindfulness Based Stress Reduction (MBSR) would improve high sensitivity C-reactive protein (hsCRP) and D-dimer in HIV+ adults, and to explore the cross-sectional and longitudinal relationships between psychological state and these markers.

## Methods

We randomized antiretroviral-untreated HIV+ adults with CD4+ counts >250 cells/µl to MBSR or an education/support control group. Baseline, 3, and 12 month measures included: Perceived Stress Scale (PSS), Beck Depression Inventory (BDI), Patient Health Questionnaire-9 (PHQ), State Trait Anxiety Inventory (STAI), and Positive and Negative Affect Scale (PANAS+/-). Data were censored for starting antiretroviral therapy during follow-up.

## Results

Of 177 participants, 132 (71 MBSR, 61 control) had complete specimen panels and were eligible for this sub-study. MBSR did not appear to have a substantial effect on change in hsCRP or D-dimer from baseline to 3, or 12 months (p>0.10), though CIs were wide. hsCRP at baseline was positively correlated with: PSS (β=0.18, p=0.034), BDI (β=0.21, p=0.014), PHQ (β=0.15, p=0.087), PANAS+/- (β=0.17, p=0.049), and STAI (β=0.19, p=0.030). hsCRP was correlated with BMI (β=0.25, p=0.004). After controlling for BMI, age, and viral load, hsCRP remained associated with BDI (β=0.19 p=0.03) and STAI (β=0.16 p=0.065). D-dimer showed no substantial baseline correlation with any scale (β<0.1, p>0.5). No substantial longitudinal relationships were found between change in hsCRP or D-dimer and change in any psychological measure (β<0.12, p>0.2).

## Conclusion

MBSR did not appear to substantially improve hsCRP or D-dimer. Correlations between hsCRP and psychological measures were in hypothesized directions. The observation that hsCRP was associated with depression in multivariate analysis suggests a causal association between these processes. Interventional studies aimed at reducing inflammation, or improving mood, are needed to clarify this association and to identify future therapeutic strategies.

